# Phenotypic variation of erythrocyte linker histone H1.c in a pheasant (*Phasianus colchicus* L.) population

**DOI:** 10.1590/S1415-47572010000300016

**Published:** 2010-09-01

**Authors:** Andrzej Kowalski, Jan Pa³yga, Ewa Górnicka-Michalska, Zenon Bernacki, Marek Adamski

**Affiliations:** 1 Department of Biochemistry and Genetics, Jan Kochanowski University, KielcePoland; 2 Department of Poultry Breeding, University of Technology and Life Sciences, BydgoszczPoland

**Keywords:** linker histones, H1.c subtype, allelic variants, electrophoresis

## Abstract

Our goal was to characterize a phenotypic variation of the pheasant erythrocyte linker histone subtype H1.c. By using two-dimensional polyacrylamide gel electrophoresis three histone H1.c phenotypes were identified. The differently migrating allelic variants H1.c1 and H1.c2 formed either two homozygous phenotypes, c1 and c2, or a single heterozygous phenotype, c1c2. In the pheasant population screened, birds with phenotype c2 were the most common (frequency 0.761) while individuals with phenotype c1 were rare (frequency 0.043).

Linker histones, also known as H1 histones, are members of a protein family composed of small (~21.5 kDa) and abundant (~0.8 H1/nucleosome) basic proteins, located in the eukaryotic chromatin (Woodcock *et al.*, 2006). In the past, they were mainly recognized as structural components involved in stabilizing nucleosomal arrays and folding chromatin fiber into more compacted states (Widom, 1998; Hansen, 2002;). However, a contribution of histone H1 to other nuclear events, such as specific gene regulation (Lee *et al.*, 2004), DNA methylation (Fan *et al.*, 2005) or cell cycle disruption (Sancho *et al.*, 2008), was also demonstrated. As highly mobile proteins, the members of the histone H1 family can compete with other structural (Catez *et al.*, 2004) and regulatory proteins (Kim *et al.*, 2008) to change their activity in a dynamic network of chromatin interactions (Bustin *et al.*, 2005).

Higher eukaryotes have at least six somatic histone H1 subtypes (van Holde, 1989), encoded by separate genes intermingled with core histone genes (Nakayama *et al.*, 1993). H1 histone variants usually differ in amino acid sequence in less structured N-terminal and C-terminal domains and occasionally in highly conservative and structured central globular domain (Ponte *et al.*, 1998). The avian family of somatic linker histones, composed of at least six to seven non-allelic subtypes that can be identified according to the rate of their electrophoretic migration in polyacrylamide gels (Palyga, 1991a), may differ in the number of components in different species. While faster moving histone H1 subtypes H1.c, H1.c' and H1.d were found to be present in every species tested, the slower migrating subtypes H1.a', H1.b' and H1.z were occasionally missing. For example, histone H1 subtype H1.z that is present in ducks (Palyga *et al.*, 1993), quails (Palyga, 1998a) and many other species (Palyga, 1991a) has not been observed in chickens (Górnicka-Michalska *et al.*, 2006) and partridges (Kowalski *et al.*, 2008). A comparison of the gel patterns of avian H1 histones demonstrated that species-specific components possessed slightly higher molecular weights, and hence migrated slower, than faster moving subtypes that tended to arrange in a triangle-shaped pattern (Palyga, 1991a). In addition, a polymorphic variation was found to be typical for histone subtypes H1.a (Kowalski *et al.*, 1998; Palyga, 1998a; Górnicka-Michalska *et al.*, 2006), H1.a' (Kowalski *et al.*, 2008), H1.b (Palyga, 1998a; Palyga *et al.*, 2000;) and H1.z (Palyga *et al.*, 1993; Palyga, 1998a; Kowalski *et al.*, 2004) in several avian species, whereas the histone subtypes H1.c, H1.c' and H1.d were relatively invariant in all species tested so far.

In this study, we show that pheasant erythrocyte histone H1.c is a heterogeneous protein with two allelic variants, H1.c1 and H1.c2, which occur as homozygous, c1 and c2, and heterozygous, c1c2, phenotypic combinations.

The study was carried out using a group of 46 pheasants (*Phasianus colchicus* L.) bred at the Department of Poultry Breeding of the University of Technology and Life Sciences at Bydgoszcz, Poland. Erythrocytes were isolated from a cell suspension consisting of 1/3 of whole blood and 2/3 of SSC solution (0.15 M NaCl, 0.015 M sodium citrate) by triple washing with SSC. Erythrocyte nuclei were prepared by lysis with a 3% saponin solution buffered with 0.1 M sodium phosphate, pH 7.0. After washing the nuclear pellet several times with 0.9% NaCl, the total acid-soluble fraction containing mainly histone H1 proteins was isolated by double extraction of the crude nuclear pellet with perchloric acid, first with a 1 M and then with a 0.5 M solution. The protein was precipitated with trichloroacetic acid, then the pellet was washed, first with acetone acidified with HCl and finally with pure acetone, and air-dried.

Electrophoresis samples, prepared by dissolving 1-mg aliquots of a total histone H1 protein preparation in 200 μL of a solution containing 8 M urea, 0.9 M acetic acid and 10% 2-mercaptoethanol, were submitted to two-dimensional polyacrylamide gel electrophoresis. First, the total protein was resolved in a 15% acrylamide gel containing 8 M urea and 0.9 M acetic acid in the first dimension, and then in a 13.5% acrylamide gel prepared with 0.1% sodium dodecylsufate (SDS) in the second dimension, according to the detailed description of Palyga (1991b). The gels were stained with Coomassie Blue R-250 and images taken by means of a Doc-Print II gel documentation system (Vilber Lourmat).

Total preparations of pheasant histone H1, containing H1.c and other H1 subtypes, obtained from saponin-lysed erythrocyte nuclei by perchloric acid extraction, were separated both by one-dimensional and two-dimensional polyacrylamide gel electrophoresis (Figure 1A). A comparison of the electrophoretic mobility of a faintly stained band of histone H1.c (band c2) of the pheasant population in the acetic acid-urea polyacrylamide gel (Figure 1A) revealed that it was missing in some individuals, while the histone H1.c spots in the two-dimensional gel were clearly present in all individuals (Figure 1B). Therefore, it appears that pheasant erythrocyte histone H1.c is a heterogeneous protein with a presumed polymorphic variation, while the other histone H1 non-allelic subtypes, H1.a, H1.b, H1.b', H1.c' and H1.d, are monomorphic proteins (Figure 1). The electrophoretic migration of H1.c is determined by the mobility of its allelic complements, H1.c1 and H1.c2, in a particular type of gel. A slow isoform, H1.c1, migrated along with the subtype H1.b in the first dimension and was positioned further away from the nearest moving subtype H1.d in the second dimension, whereas the fast isoform, H1.c2, migrated slightly below the subtype H1.b' in the first dimension and in a direct vicinity of subtype H1.d in the second dimension (Figure 1C). The histone H1.c heterogeneity detected in the acetic acid-urea gel may result from differences in a net charge between the allelic isoforms H1.c1 and H1.c2. Previously, by using acid-urea polyacrylamide gel, we disclosed the polymorphisms of histone H1.a in ducks (Kowalski *et al.*, 1998) and chickens (Górnicka-Michalska *et al.*, 2006). In both cases, two allelic variants, H1.a1 and H1.a2, formed three phenotypes, a1, a2 and a1a2, which appeared to be differently distributed in the avian breeds and/or genetic groups tested. Most of the duck and chicken specimens were found to possess an abundant phenotype a1, which was the only form of histone H1.a in some avian flocks. A rare phenotype, a2, was present at a frequency below 10% in several duck groups (Kowalski *et al.*, 1998) and has not been detected in any chicken population (Górnicka-Michalska *et al.*, 2006). In the latter, the structural properties of the allelic isoform H1.a2 were analyzed using preparations obtained from homozygous a2 individuals using progeny of purpose-mated heterozygous parents (unpublished). In the pheasant population tested, the allelic isoforms of histone H1.c were found to be arranged into three phenotypes. In particular, the isoforms H1.c1 and H1.c2 were constituents of homozygous phenotypes c1 and c2, respectively, or combined together to constitute the heterozygous phenotype c1c2 (Figure 1). Among the 46 pheasants tested, the majority (35 individuals) was represented by homozygotes for isoform H1.c2 (frequency = 0.761), while the heterozygotes (9 individuals) with both isoforms H1.c1 and H1.c2 (frequency = 0.195) and homozygotes for isoform H1.c1 (frequency = 0.043) were minority. Thus, in the pheasant population tested, the prevailing allele *c*^2^ (frequency = 0.858) was found to occur at a frequency more than six times higher than that of the rare allele *c*^1^ (frequency = 0.141) (Table 1).

**Figure 1:**
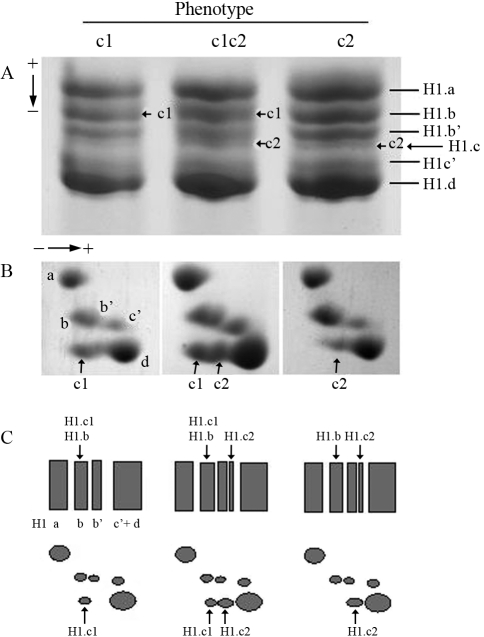
Phenotypic variation of pheasant histone H1.c in one-dimensional (A) and two-dimensional (B) gels. Identified phenotypes of histone H1.c (c1, c2 and c1c2) are composed of a single band (A) or spot (B) of allelic isoform H1.c1 (homozygous phenotype c1) or allelic isoform H1.c2 (homozygous phenotype c2), or a double band (A) or spot (B) containing both isoforms H1.c1 and H1.c2 (heterozygous phenotype c1c2). In the acetic acid-urea gel, the isoform H1.c1, comigrating with subtype H1.b, moved slower than the fast isoform H1.c2, migrating slightly below subtype H1.b', while both variants exhibited the same rate of electrophoretic migration (and presumably similar molecular weights) in the SDS gel, but were differently located in relation to the H1.d spot (C).

Various combinations of allelic components of the polymorphic histone H1 subtypes have been identified in several avian species, including ducks (Palyga *et al.*, 1993; Kowalski *et al.*, 1998), quails (Palyga, 1998a), chickens (Górnicka-Michalska *et al.*, 2006), and partridges (Kowalski *et al.*, 2008). Due to differences, either in net charge and/or in molecular weight, the allelic isoforms could be distinguished based on their electrophoretic migration in polyacrylamide gels. Usually, two or three allelic variants which might form three or six phenotypes, respectively, were detected in the populations tested. For example, polymorphic duck subtypes H1.a (Kowalski *et al.*, 1998) and H1.z (Palyga *et al.*, 1993), each composed of two allelic isoforms, formed three phenotypes, while Peking duck histone H1.b (Palyga *et al.*, 2000) and Muscovy duck histone H1.z (Kowalski *et al.*, 2004), with three allelic isoforms each, were combined to form six phenotypes.

A variation in the frequency of phenotypes and alleles among polymorphic histone H1 subtypes was observed in several avian populations (Palyga *et al.*, 1993; Kowalski *et al.*, 2004; Górnicka-Michalska *et al.*, 2006; Kowalski *et al.*, 2008). The allele frequency was also found to correlate with a selection aimed at improving some usable traits in poultry breeding (Palyga, 1998b; Palyga *et al.*, 2000). Moreover, we observed (unpublished data) a tendency to decrease heterozygosity, almost by half for subtype H1.b, and more than five times for subtype H1.z, between quails selected for a high cholesterol content in the eggs (Baumgartner *et al.*, 2007) and an unselected quail population. These and other selection results (Palyga, 1998b) seem to suggest that some histone H1 polymorphic subtypes may affect the mechanisms and/or processes underlying the breeding traits and the quality of animal products. As even slight changes in the histone H1 primary structure can influence chromatin properties (Bharath *et al.*, 2003; Hendzel *et al.*, 2004), it seems that a small structural difference between histone H1 allelic variants may modify their binding to chromatin, possibly modulating chromatin remodeling and regional gene regulation.

## Figures and Tables

**Table 1:** Phenotype and allele frequencies of pheasant erythrocyte histone H1.c.

Histone H1.c phenotype	Nº of individuals	Phenotype frequency	Allele frequency	
			*c*^1^	*c*^2^
c1	2	0.043		
c2	35	0.761	0.141	0.858
c1c2	9	0.195		
